# Oxylipin Profiling in Selected Brown and Red Algae: Detection of Heterobicyclic Oxylipins, Plasmodiophorols and Ectocarpins in Phaeophyceae

**DOI:** 10.3390/md24010008

**Published:** 2025-12-23

**Authors:** Yana Y. Toporkova, Elena O. Smirnova, Oksana S. Belous, Tatiana M. Iljina, Natalia V. Lantsova, Svetlana S. Gorina, Alexander N. Grechkin

**Affiliations:** 1 Kazan Institute of Biochemistry and Biophysics, FRC Kazan Scientific Center of RAS, P.O. Box 261, 420111 Kazan, Russia; 2 A.V. Zhirmunsky National Scientific Center of Marine Biology, Far Eastern Branch, Russian Academy of Sciences, 690041 Vladivostok, Russia

**Keywords:** lipoxygenase cascade, oxylipins, plasmodiophorols, ectocarpins, hydroperoxide bicyclase, allene oxide synthase, epoxyalcohol synthase, brown algae, red algae

## Abstract

GC-MS oxylipin profiling of brown and red algal thalli was performed. Brown algae (*Fucus distichus* and *Alaria esculenta*) were collected from the Barents Sea coastline nearby Teriberka, Murmansk region, Kola Peninsula, Russia, while other brown and red algae were sourced from the Pacific coast of the Russian Far East. Triols and δ-ketols (epoxyalcohol synthase products) were found in most brown and red algae. Several Heterokontophyta and Rhodophyta species possessed α-ketols (products of allene oxide synthase) and related *vic*-diols. Plasmodiophorols and ectocarpins (hydroperoxide bicyclase (HPB) products) were found only in brown algae from the Ectocarpales, Fucales, and Laminariales orders, not in brown algae from the Desmarestiales or Dictyotales orders, or in any red algae. Therefore, plasmodiophorol A and other HPB products could be used as chemotaxonomic markers for the classification of the separate orders of algae within Heterokontophyta. The in vitro incubations of *F. distichus* thalli with linoleic and α-linolenic acid resulted in the formation of α-ketols and the hydroperoxide bicyclase product, plasmodiophorol A.

## 1. Introduction

In many aerobic organisms, the lipoxygenase cascade is the main source of diverse bioactive oxylipins, which are products of the oxidative metabolism of unsaturated fatty acids [1,2,3]. First and foremost, in mammals, eicosanoids play a crucial role in regulating essential physiological functions [1]. In plants, lipoxygenases and cytochromes P450 of the CYP74 family are the key enzymes for the biosynthesis of regulatory oxylipins [2,3,4]. The CYP74 family includes allene oxide synthase (AOS) [4,5], hydroperoxide lyase (HPL) [6,7,8], divinyl ether synthase (DES) [9,10], and epoxyalcohol synthase (EAS) [11,12], which are the primary enzymes of the lipoxygenase cascade in plants. These enzymes produce α-ketols (AOS products), aldehydes and aldo acids (HPL products), divinyl ethers (DES products), and epoxyalcohols (EAS products). These substances are frequently found outside of the kingdom of plants. For example, the brown alga *Ectocarpus siliculosus* [11] was found to contain epoxyalcohol synthase EsEAS (CYP5164B1), and the brown alga *Laminaria sinclairii* [13] and the red alga *Polyneura latissima* were found to contain divinyl ethers [14].

As a whole, the marine organisms possess a high diversity of oxylipins [15,16]. In particular, some species of brown and red algae possess unique heterobicyclic oxylipins, such as hybridalactone [17], ecklonialactones [18,19,20], their haloid derivatives [21], egregiachlorides [22], agardhilactone [23], cymatherol, cymatherolactone [24], and cymathere ethers [25]. However, until recently, the pathways and enzymes involved in the biosynthesis of most of these natural products were not revealed. The key enzyme controlling the biosynthesis of such compounds, hydroperoxide bicyclase EsHPB (CYP5164A3) of *Ectocarpus siliculosus*, was detected recently [26]. The related PbHPB (CYP50918A1) enzyme was detected [27] in another member of the SAR (stramenopiles, alveolates, and rhizaria) eukaryote clade, the cercozoan *Plasmodiophora brassicae*, the cabbage pathogen that causes the clubroot disease. Both enzymes specifically converted the 13(*S*)-hydroperoxide of α-linolenic acid and 15(*S*)-hydroperoxide of eicosapentaenoic acid to the novel ecklonialactone-related products named plasmodiophorols A-C and ectocarpins A, C, and D, respectively [26,27]. In this study, we analyzed the thalli of some phylogenetically distant species of brown and red algae for the presence of plasmodiophorols and ectocarpins.

## 2. Results

### 2.1. GC-MS Profiling of Oxylipins from Thalli of Fucus distichus

**Figure 1:**
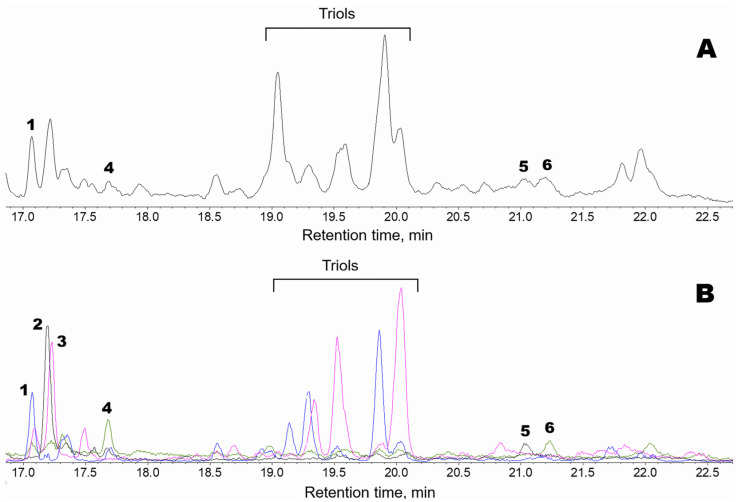
GC-MS profiles of the endogenous oxylipins (Me/TMS) from thalli of *Fucus distichus*. (**A**), Total ion current (TIC) chromatogram. (**B**), Superimposed SIC chromatograms of the selected ion currents at *m*/*z* 171 (black), *m*/*z* 173 (pink), *m*/*z* 259 (blue), and *m*/*z* 131 (green). The structural formulae of products are present in the Figure 2: **1**, 9,10-dihydroxy-12-octadecenoic acid; **2**, α-ketol 12-oxo-13-hydroxy-9,15-octadecadienoic acid; **3**, 12,13-dihydroxy-9-octadecenoic acid; **4**, plasmodiophorol A; **5**, 14,15-dihydroxy-5,8,11,17-eicosatetraenoic acid; **6**, plasmodiophorol C.

**Figure 2:**
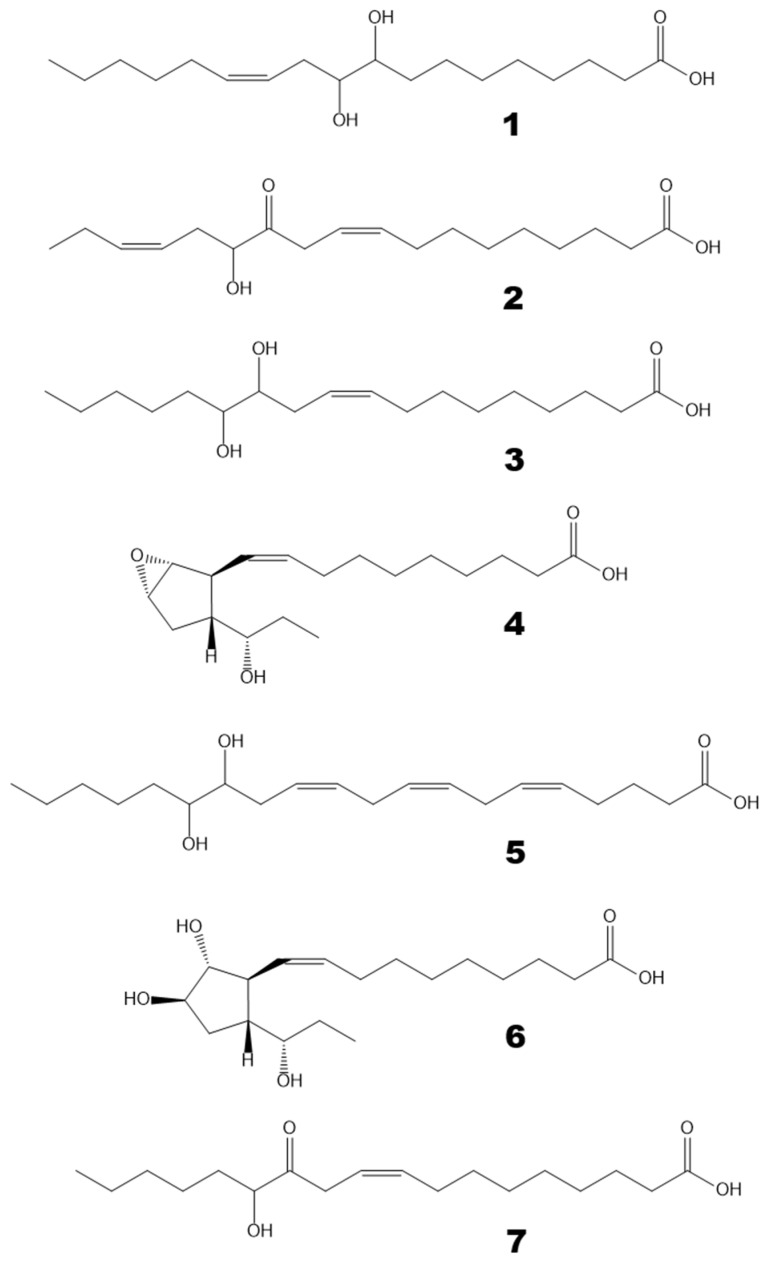
The structural formulae of oxylipins detected in *F. distichus* thalli. **1**, 9,10-dihydroxy-12-octadecenoic acid; **2**, α-ketol 12-oxo-13-hydroxy-9,15-octadecadienoic acid; **3**, 12,13-dihydroxy-9-octadecenoic acid; **4**, plasmodiophorol A; **5**, 14,15-dihydroxy-5,8,11,17-eicosatetraenoic acid; **6**, plasmodiophorol C; **7**, α-ketol 12-oxo-13-hydroxy-9-octadecenoic acid.

Oxylipins (Me/TMS) extracted from thalli of *Fucus distichus* L. were subjected to GC-MS profiling. The obtained results are presented in Figure 1. The structural formulae are shown in Figure 2. The profile, in addition to the prominent hydroxy fatty acids (Me/TMS), such as (9*Z*,11*E*,15*Z*)-13-hydroxy-9,11,15-octadecatrienoic acid (13-HOT), (9*Z*,11*E*)-13-hydroxy-9,11-octadecadienoic acid (13-HOD), and (10*E*,12*Z*)-9-hydroxy-10,12-octadecadienoic acid (9-HOD), also contained triols, δ-ketols, and compounds **1**–**5**.

The mass spectrum of compound **1** (Me/TMS, peak 1 with RT 17.05 min, Figure 1) possessed M^+^ at *m*/*z* 472 (1%), [M − MeO]^+^ at *m*/*z* 441 (1%), [M − C11/C18]^+^ at *m*/*z* 361 (4%), [361 − TMSOH]^+^ at *m*/*z* 271 (76%), [M − C10/C18]^+^ at *m*/*z* 259 (66%), *m*/*z* 243 (11%), [M − C1/C9]^+^ at *m*/*z* 213 (37%), *m*/*z* 167 (9%), *m*/*z* 155 (84%), *m*/*z* 129 (37%), *m*/*z* 109 (33%), and [SiMe_3_]^+^ at *m*/*z* 73 (100%). Complementary fragments at *m*/*z* 213 and *m*/*z* 259 indicated the *vic*-diol function at C9,C10. Hydrogenation of product **1** (Me) over PtO_2_ followed by trimethylsilylation resulted in 9,10-dihydrostearic acid (Me/TMS). As judged by its mass spectrum: [M − Me]^+^ at *m*/*z* 459 (0.2%), [M − MeO]^+^ at *m*/*z* 443 (2%), [M − C10/C18 + TMS]^+^ at *m*/*z* 332 (66%), [M − C10/C18]^+^ at *m*/*z* 259 (85%), *m*/*z* 243 (11%), [M − C1/C9]^+^ at *m*/*z* 215 (85%), *m*/*z* 155 (84%), [Me_3_Si–O^+^=SiMe_2_]^+^ at *m*/*z* 147 (24%), *m*/*z* 109 (29%), *m*/*z* 95 (9%), *m*/*z* 83 (31%), and [SiMe_3_]^+^ at *m*/*z* 73 (100%). Overall, the data indicated that compound **1** is 9,10-dihydroxy-12-octadecenoic acid. One should note that *vic*-diol **1** was represented by *threo* and *erythro* diastereomers at a ratio of ca. 4:1. The *erythro* isomer (RT 17.31) had the identical mass spectrum.

Compound **2** (Me/TMS, peak 2 with RT 17.17 min, Figure 1) exhibited the distinguishing patterns of α-ketol, 12-oxo-13-hydroxy-9,15-octadecadienoic acid (Me/TMS), as seen from its mass fragmentation patterns [28,29]: [M − Me]^+^ at *m*/*z* 381 (1%), [M − MeCH_2_CH=CHCH_2_CH(OTMS)C=O + TMS]^+^ at *m*/*z* 270 (15%), [M − C1/C12]^+^ at *m*/*z* 171 (100%), *m*/*z* 129 (45%), [Me_3_Si–O^+^=CH_2_] at *m*/*z* 103 (19%), *m*/*z* 81 (44%), *m*/*z* 75 (29%), and [SiMe_3_]^+^ at *m*/*z* 73 (100%). The NaBH_4_ reduction in compound **2**, followed by methylation and trimethylsilylation, yielded the corresponding *threo* and *erythro vic*-diols, 12,13-dihydroxy-9,15-octadecadienoic acid (Me/TMS): [M − Me]^+^ at *m*/*z* 455 (0.3%), [M − MeO]^+^ at *m*/*z* 439 (1%), [M − MeCH_2_CH=CHCH_2_ − TMSOH]^+^ at *m*/*z* 311 (37%), [M − MeCH_2_CH=CHCH_2_CHOTMS]^+^ at *m*/*z* 299 (19%), [M − C1/C11]^+^ at *m*/*z* 273 (15%), [M − MeCH_2_CH=CHCH_2_CH(OTMS)CHOTMS + TMS]^+^ at *m*/*z* 270 (10%), [273 − TMSOH]^+^ at *m*/*z* 183 (25%), [M − C1/C12]^+^ at *m*/*z* 171 (28%), [Me_3_Si–O^+^=SiMe_2_]^+^ at *m*/*z* 147 (26%), *m*/*z* 129 (59%), [Me_3_Si–O^+^=CH_2_] at *m*/*z* 103 (28%), *m*/*z* 81 (27%), and [SiMe_3_]^+^ at *m*/*z* 73 (100%). The sequential NaBH_4_ reduction and catalytic hydrogenation of compound **2** yielded the *vic*-diol 12,13-dihydroxystearic acid: [M − Me]^+^ at *m*/*z* 459 (0.1%), [M − MeO]^+^ at *m*/*z* 443 (2%), [M − Me(CH_2_)_4_CHOTMS + TMS]^+^ at *m*/*z* 374 (5%), [M − Me(CH_2_)_4_CHOTMS]^+^ at *m*/*z* 301 (63%), *m*/*z* 285 (5%), *m*/*z* 197 (15%), [M − C1/C12]^+^ at *m*/*z* 173 (100%), *m*/*z* 161 (23%), [Me_3_Si–O^+^=SiMe_2_]^+^ at *m*/*z* 147 (19%), *m*/*z* 129 (15%), [Me_3_Si–O^+^=CH_2_] at *m*/*z* 103 (25%), *m*/*z* 83 (31%), and [SiMe_3_]^+^ at *m*/*z* 73 (79%). To summarize, the data affirmed that compound **2** is α-ketol, 12-oxo-13-hydroxy-9,15-octadecadienoic acid.

The mass spectrum of compound **3** (Me/TMS, peak 3 with RT 17.23 min, Figure 1) possessed [M − Me]^+^ at *m*/*z* 457 (0.3%), [M − MeO]^+^ at *m*/*z* 441 (2%), [441 − TMSOH]^+^ at *m*/*z* 351 (0.4%), [M − Me(CH_2_)_4_CHOTMS]^+^ at *m*/*z* 299 (24%), [M − C1/C11]^+^ at *m*/*z* 275 (30%), [M − Me(CH_2_)_4_CH(OTMS)CHOTMS + TMS]^+^ at *m*/*z* 270 (12%), [275 − TMSOH]^+^ at *m*/*z* 185 (31%), [M − C1/C12]^+^ at *m*/*z* 173 (56%), *m*/*z* 159 (10%), *m*/*z* 149 (12%), [Me_3_Si–O^+^=SiMe_2_]^+^ at *m*/*z* 147 (22%), *m*/*z* 129 (16%), [Me_3_Si–O^+^=CH_2_] at *m*/*z* 103 (24%), *m*/*z* 95 (22%), *m*/*z* 83 (23%), and [SiMe_3_]^+^ at *m*/*z* 73 (100%). Complementary fragments at *m*/*z* 173 and *m*/*z* 299 indicated the *vic*-diol function at the 12,13 position. Catalytic hydrogenation of compound **3** yielded the *vic*-diol 12,13-dihydroxystearic acid, described in the preceding paragraph. Overall, the MS data showed that compound **3** is the *vic*-diol, 12,13-dihydroxy-9-octadecenoic acid. It should be noted that *vic*-diol **3** was composed of *threo* and *erythro* diastereomers at a ratio of ca. 4:1.

The mass spectrum of compound **4** (Me/TMS, peak 4 with RT 17.65 min, Figure 1) showed [M − Me]^+^ at *m*/*z* 381 (0.2%), [M − Et]^+^ at *m*/*z* 367 (0.2%), [M − EtCHOTMS + TMS]^+^ at *m*/*z* 338 (1%), *m*/*z* 291 (1%), *m*/*z* 249 (1%), [338 − TMSOH]^+^ at *m*/*z* 248 (2%), *m*/*z* 181 (3%), *m*/*z* 157 (4%), *m*/*z* 142 (2%), [EtCH=O^+^–SiMe_3_] at *m*/*z* 131 (100%), *m*/*z* 119 (10%), *m*/*z* 105 (13%), *m*/*z* 91 (16%), and [SiMe_3_]^+^ at *m*/*z* 73 (100%). The base peak at *m*/*z* 131 indicated the presence of a 1-(TMS-oxy)propyl substituent. The spectrum matched that of plasmodiophorol A, the substituted 6-oxabicyclo[3.1.0]hexane [27]. Hydrogenation of product **4** over PtO_2_ resulted in a saturated analog whose spectrum (Me/TMS) possessed M^+^ at *m*/*z* 398 (0.1%), [M − Me]^+^ at *m*/*z* 383 (0.3%), [M − Et]^+^ at *m*/*z* 369 (0.6%), [383 − MeOH]^+^ at *m*/*z* 351 (0.1%), *m*/*z* 341 (0.4%), [M − EtCHOTMS + TMS]^+^ at *m*/*z* 340 (0.3%), [383 − TMSOH]^+^ at *m*/*z* 293 (1%), [369 − TMSOH]^+^ at *m*/*z* 279 (0.5%), [279 − MeOH]^+^ at *m*/*z* 247 (2%), *m*/*z* 201 (3%), *m*/*z* 157 (4%), *m*/*z* 155 (20%), [EtCH=O^+^–SiMe_3_] at *m*/*z* 131 (100%), *m*/*z* 121 (10%), *m*/*z* 109 (10%), *m*/*z* 107 (13%), *m*/*z* 93 (9%), *m*/*z* 81 (17%), *m*/*z* 75 (20%), and [SiMe_3_]^+^ at *m*/*z* 73 (67%). Thus, the hydrogenation confirmed the structure of substituted 6-oxabicyclo[3.1.0]hexane and the presence of one double bond in parent compound **4**. Overall, the data indicated that compound **4** is plasmodiophorol A, (9*Z*)-10-{(1′*R*,2′*S*,3′*R*,5′*S*)-3′-[(1″*S*)-1″-hydroxypropyl]-6′-oxabicyclo[3.1.0]hex-2′-yl}-9-decenoic acid [27].

The mass spectrum of compound **5** (Me/TMS, peak 5 with RT 21.04 min, Figure 1) showed [M − Me]^+^ at *m*/*z* 405 (0.2%), [M − MeCH_2_CH=CHCH_2_CH(OTMS)C=O + TMS]^+^ at *m*/*z* 294 (5%), [M − C1/C14]^+^ at *m*/*z* 171 (73%), *m*/*z* 129 (85%), [Me_3_Si–O^+^=CH_2_] at *m*/*z* 103 (14%), *m*/*z* 81 (43%), *m*/*z* 75 (29%), and [SiMe_3_]^+^ at *m*/*z* 73 (100%). Mass fragmentation patterns advocated the structure of α-ketol, 14-oxo-15-hydroxy-5,8,11,17-eicosatetraenoic acid (Me/TMS) for compound **5**. The NaBH_4_ reduction in compound **5** followed by methylation and trimethylsilylation yielded the corresponding *threo* and *erythro vic*-diols, as judged by their mass spectra: M^+^ at *m*/*z* 494 (0.6%), [M − Me]^+^ at *m*/*z* 479 (0.3%), [M − MeCH_2_CH=CHCH_2_ − TMSOH]^+^ at *m*/*z* 335 (2%), [M − MeCH_2_CH=CHCH_2_CHOTMS]^+^ at *m*/*z* 323 (0.4%), [M − C1/C13]^+^ at *m*/*z* 273 (5%), [M − MeCH_2_CH=CHCH_2_CH(OTMS)CHOTMS + TMS]^+^ at *m*/*z* 294 (2%), [335 − TMSOH]^+^ at *m*/*z* 245 (4%), *m*/*z* 213 (4%), *m*/*z* 201 (4%), [273 − TMSOH]^+^ at *m*/*z* 183 (19%), [M − C1/C14]^+^ at *m*/*z* 171 (20%), *m*/*z* 159 (10%), [Me_3_Si–O^+^=SiMe_2_]^+^ at *m*/*z* 147 (17%), *m*/*z* 129 (34%), [Me_3_Si–O^+^=CH_2_] at *m*/*z* 103 (21%), *m*/*z* 93 (14%), *m*/*z* 81 (19%), and [SiMe_3_]^+^ at *m*/*z* 73 (100%). The mass spectrum indicated the structure of 14,15-dihydroxy-5,8,11,17-eicosatetraenoic acid (Me/TMS) for the product of NaBH_4_ reduction in compound **5**. The sequential NaBH_4_ reduction and catalytic hydrogenation of product **5** resulted in the formation of the *vic*-diol 14,15-dihydroxyeicosanoic acid: [M − MeO]^+^ at *m*/*z* 471 (2%), [M − Me(CH_2_)_4_CHOTMS + TMS]^+^ at *m*/*z* 402 (5%), [M − Me(CH_2_)_4_CHOTMS]^+^ at *m*/*z* 329 (66%), *m*/*z* 313 (3%), *m*/*z* 207 (15%), *m*/*z* 189 (23%), [M − C1/C14]^+^ at *m*/*z* 173 (100%), *m*/*z* 159 (10%), [Me_3_Si–O^+^=SiMe_2_]^+^ at *m*/*z* 147 (26%), *m*/*z* 129 (18%), [Me_3_Si–O^+^=CH_2_] at *m*/*z* 103 (28%), *m*/*z* 83 (39%), and [SiMe_3_]^+^ at *m*/*z* 73 (92%). Altogether, the data affirmed that compound **5** is α-ketol, 14-oxo-15-hydroxy-5,8,11,17-eicosatetraenoic acid.

Compound **6** (Me/TMS, peak 6 with RT 21.23 min, Figure 1), similarly to plasmodiophorol A (**4**), exhibited a prominent distinguishing fragment at *m*/*z* 131 in its mass spectrum. In addition, the mass spectrum of compound **6** (Me/TMS) possessed [M − Et − MeOH]^+^ at *m*/*z* 497 (2%), [M − Me − TMSOH]^+^ at *m*/*z* 453 (6%), [M − Et − TMSOH]^+^ at *m*/*z* 439 (1%), [M − 2TMSOH]^+^ at *m*/*z* 3787 (4%), [439 − TMSOH]^+^ at *m*/*z* 349 (3%), *m*/*z* 337 (2%), [M − C13/C18]^+^ at *m*/*z* 297 (8%), *m*/*z* 288 (3%), [337 − TMSO]^+^ at *m*/*z* 248 (8%), *m*/*z* 159 (7%), *m*/*z* 157 (3%), *m*/*z* 151 (6%), [Me_3_Si–O^+^=SiMe_2_]^+^ at *m*/*z* 147 (17%), [M − C1/C15]^+^ at *m*/*z* 131 (100%), *m*/*z* 129 (10%), [Me_3_Si–O^+^=CH_2_] at *m*/*z* 103 (8%), *m*/*z* 95 (12%), *m*/*z* 93 (17%), and [SiMe_3_]^+^ at *m*/*z* 73 (55%). The spectrum matched that for 2,3-dihydroxycyclopent-1-yl carbinol, called plasmodiophorol C [27]. Catalytic hydrogenation converted it into a 9,10-dihydro analog, exhibiting a highly characteristic mass spectrum: *m*/*z* 477 (2%), *m*/*z* 448 (2%), [M − MeO − TMSOH]^+^ at *m*/*z* 439 (1%), [M − Et − 2TMSOH]^+^ at *m*/*z* 351 (12%), [M − EtCHOTMS − TMSOH]^+^ at *m*/*z* 339 (4%), *m*/*z* 247 (2%), *m*/*z* 183 (2%), *m*/*z* 159 (7%), [Me_3_Si–O^+^=SiMe_2_]^+^ at *m*/*z* 147 (6%), [M − C1/C15]^+^ at *m*/*z* 131 (100%), *m*/*z* 121 (4%), *m*/*z* 119 (4%), *m*/*z* 95 (7%), *m*/*z* 93 (8%), *m*/*z* 81 (6%), *m*/*z* 75 (9%), and [SiMe_3_]^+^ at *m*/*z* 73 (49%). The spectrum totally corresponded to that for the 9,10-dihydro derivative of plasmodiophorol C [27]. Overall, the data confirmed the structure of plasmodiophorol C, (9*Z*)-10-{(1′*S*,2′*R*,3′*R*,5′*R*)-2′,3′-dihydroxy-5′-[(1″*S*)-1″-hydroxypropyl]cyclopentyl}-9-decenoic acid, for compound **6**.

To reveal the biosynthetic origin of oxylipins, the GC-MS profiling was conducted after preincubating *F. distichus* thalli homogenate with exogenous linoleic and α-linolenic acids. The results of these experiments are presented in the following section.

### 2.2. In Vitro Biosynthesis of Oxylipins in Thalli of Fucus distichus

**Figure 3:**
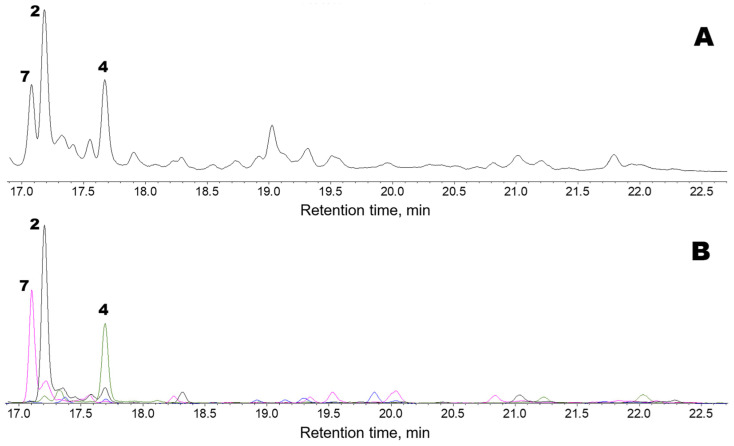
GC-MS profiles of the oxylipins (Me/TMS) observed after preincubating *F. distichus* thalli homogenate with a mixture of exogenous linoleic and α-linolenic acids. (**A**), TIC chromatogram. (**B**), Superimposed SIC chromatograms of the selected ion currents at *m*/*z* 171 (black), *m*/*z* 173 (pink), *m*/*z* 259 (blue), and m/z 131 (green). The structural formulae of products are present in the Figure 2: **2**, α-ketol 12-oxo-13-hydroxy-9,15-octadecadienoic acid; **4**, plasmodiophorol A; **7**, α-ketol 12-oxo-13-hydroxy-9-octadecenoic acid. Compound **7** (Me/TMS) was not endogenously detected. Its mass spectrum exhibited M^+^ at *m*/*z* 398 (0.1%), [M − Me]^+^ at *m*/*z* 383 (2%), [M − C12/C18 + TMS]^+^ at *m*/*z* 270 (12%), [M − C1/C12]^+^ at *m*/*z* 173 (100%), *m*/*z* 159 (3%), *m*/*z* 129 (2%), [Me_3_Si–O^+^=CH_2_] at *m*/*z* 103 (32%), *m*/*z* 83 (27%), *m*/*z* 75 (12%), and [SiMe_3_]^+^ at *m*/*z* 73 (58%). The spectrum matched that of α-ketol, 12-oxo-13-hydroxy-9-octadecenoic acid (Me/TMS), an AOS product derived from the 13(*S*)-hydroperoxide of linoleic acid (13-HPOD) via the short-lived allene oxide [30]. The NaBH_4_ reduction in compound **7**, followed by methylation and trimethylsilylation, resulted in *vic*-diol **3** (Me/TMS), which was composed of *threo* and *erythro* isomers in a ca. 2:1 ratio. This result supported the identification of compound **7** as an α-ketol. The NaBH4 reduction in compound **7**, followed by catalytic hydrogenation, methylation, and trimethylsilylation, resulted in *vic*-diol 12,13-dihydroxystearic acid (Me/TMS). Overall, the data obtained showed that compound **7** is the α-ketol, 12-oxo-13-hydroxy-9-octadecenoic acid. Thus, products **2** and **7** were α-ketols biosynthesized through the AOS pathway from 13-HPOT and 13-HPOD, respectively.

Figure 3 shows the oxylipin (Me/TMS) profile observed after preincubating *F. distichus* thalli homogenate with a mixture of exogenous linoleic and α-linolenic acids. There were three major products: compounds **7**, **2**, and **4**. Products **2** and **4** are described above. The α-ketol (**2**), 12-oxo-13-hydroxy-9,15-octadecadienoic acid, is an allene oxide synthase (AOS) product that is biosynthesized from the 13(*S*)-hydroperoxide of α-linolenic acid (13-HPOT) via allene oxide. Plasmodiophorol A (**4**) is the product of 13-HPOT conversion by hydroperoxide bicyclase (HPB).

Compound **4**, plasmodiophorol A, was also present endogenously, but its abundance was higher after the in vitro preincubation of a homogenate of *F. distichus* thalli with a mixture of the exogenous linoleic and α-linolenic acids (Figure 3) than in the profile of endogenous *F. distichus* oxylipins (Figure 1). To conclude, the major hydroperoxide-metabolizing enzymes detected in *F. distichus* homogenate were AOS and HPB.

In vitro oxylipin biosynthesis was also studied with *A. esculenta* thalli homogenate. These incubation results were similar to those obtained using *F. distichus*. Therefore, the in vitro data obtained using *A*. *esculenta* are not presented. The next section presents data on the endogenous oxylipins of *A. esculenta* and several Pacific brown and red algae species.

### 2.3. Occurrence of Oxylipins in Thalli of Some Brown and Red Algae Species

Data from the GC-MS profiling of oxylipins (Me/TMS) from selected brown and red algae are presented in Table 1. Brown algae possessed the highest oxylipin diversity. Most brown algae, except *Coccophora langsdorfii*, possessed some AOS products, namely the α-ketols (Me/TMS), 12-oxo-13-hydroxy-9,15-octadecadienoic acid (**2**), 14-oxo-15-hydroxy-5,8,11,17-eicosatetraenoic acid (**5**), and 12-oxo-13-hydroxy-9-octadecenoic acid (**7**), as well as the related *vic*-diols 9,10-dihydroxy-12-octadecenoic acid (**1**) and 12,13-dihydroxy-9-octadecenoic acid (**3**).

**Figure 4:**
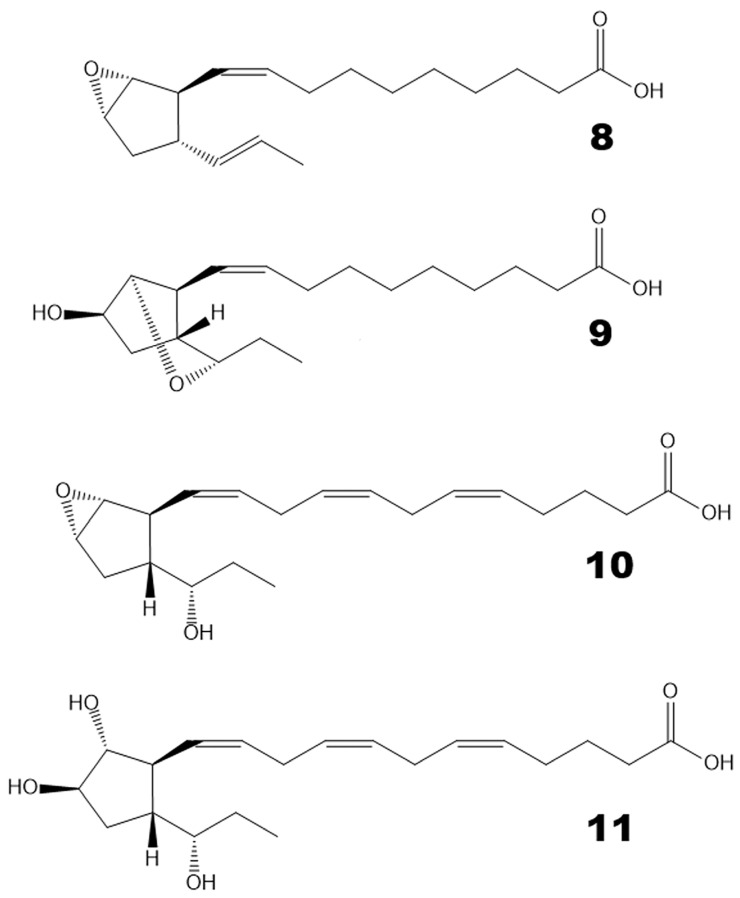
The structural formulae of additional HPB products revealed in the present report. **8**, ectocarpin A; **9**, plasmodiophorol B; **10**, ectocarpin C; **11**, ectocarpin D.

Hydroperoxide bicyclase (HPB) products represented another type of oxylipin that was abundant in brown algae but not in red algae. For example, *Undaria pinnatifida* and *Chorda filum* oxylipin profiles are presented in Supplementary Figure S1. For instance, plasmodiophorol A (**4**) was present in all studied brown algae. Plasmodiophorol C (**6**) was abundant in all brown algae, except *Alaria esculenta* and *Saccharina cichorioides*. Ectocarpin C (**10**) was revealed in almost all brown algae, except *Alaria esculenta* and *Sargassum pallidum.* Plasmodiophorol B (**9**) was relatively common. It was detected in *Punctaria plantaginea*, *Sargassum pallidum*, *Chorda filum*, *Saccharina cichorioides*, and *Undaria pinnatifida*. Ectocarpin A (**8**) was detected in *Chorda filum* and *Undaria pinnatifida*. Ectocarpin D (**11**) was found in *Undaria pinnatifida*, *Sargassum miyabei*, and *Saccharina cichorioides*. The structural formulae of compounds **8**–**11** are presented in Figure 4. The mass spectral data of HPB compounds **4**, **6**, **8**–**11** are presented in Supplementary Figures S2 and S3.

Triols were present in the thalli of the majority of studied brown and red algae (Table 1, right column). As a rule, they coexist alongside δ-ketols. The typical detected δ-ketols were, for instance, 9-oxo-13-hydroxy-11-octadecenoic and 9-hydroxy-13-oxo-10-octadecenoic acids (Me/TMS). Both triols and δ-ketols were presumably biosynthesized via the epoxyalcohol synthase (EAS) route [31]. Oxiranyl carbinols were abundant in the thalli of certain algae (Table 1). Furthermore, oxiranyl carbinols were detected in many of the studied algae at low content. These oxylipins were found in the highest abundance in separate species. The brown alga *Costaria costata* and the red alga *Dasysiphonia sessilis* possessed a high content of oxiranyl carbinol, 9,10-epoxy-11-hydroxy-12-octadecenoic acid (Me/TMS). Its mass spectrum showed a prominent distinguishing fragment [M − C1/C10]^+^ at *m*/*z* 199. *Punctaria plantaginea* and *Sargassum pallidum*, two brown algae, were found to be rich in another oxiranyl carbinol, 11-hydroxy-12,13-epoxy-9-octadecenoic acid (Me/TMS). Its mass spectrum exhibited a diagnostic fragment [M − C12/C18]^+^ at *m*/*z* 285. The biosynthetic origin of detected oxylipins and their importance as chemotaxonomic markers are discussed below.

## 3. Discussion

**Figure 5:**
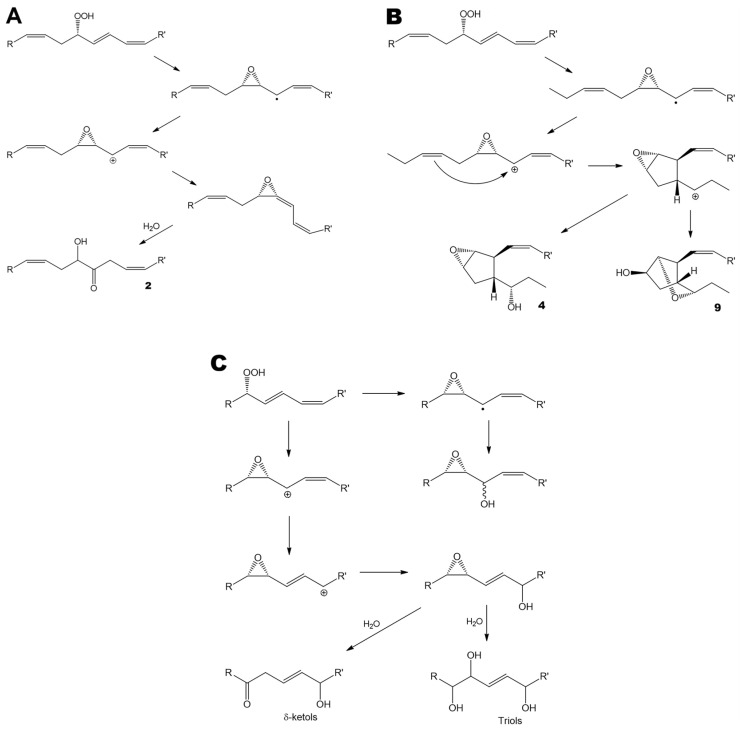
The major oxylipin biosynthetic pathways revealed in the present study. (**A**), allene oxide synthase pathway. (**B**), hydroperoxide bicyclase pathway. (**C**), epoxyalcohol synthase pathway. (**A**,**B**), R = ethyl, R’ = (CH_2_)_7_COOMe, or vice versa. (**C**), R = ethyl, R’ = (CH_2_)_7_COOMe. All conversions are enzymatic. The only exceptions are the hydrolysis of the short-lived allene oxide (Figure 5A, bottom) and the hydrolysis of oxiranyl vinyl carbinol (Figure 5C, bottom).

The obtained data demonstrated the presence of oxylipins in all studied species of brown and red algae. Most brown algae exhibited a particularly high diversity of oxylipins. This diversity encompasses the products of AOS (α-ketols **2**, **5**, and **7**), HPB (**4**, **6**, **8**, **9**, and **10**), and EAS routes (Figure 5A, 5B, and 5C, respectively). The latter group of oxylipins included epoxyalcohols (oxiranyl carbinols), δ-ketols, and triols (Figure 5C). Thomas et al. [31] demonstrated that δ-ketols, along with triols, are the products of oxiranyl vinyl carbinol hydrolysis. The oxiranyl vinyl carbinols themselves were not detected in algae, presumably because of their relatively short lifetime. These epoxyalcohols undergo hydrolysis to form stable triols and δ-ketols (Figure 5C) [31].

**Figure 6:**
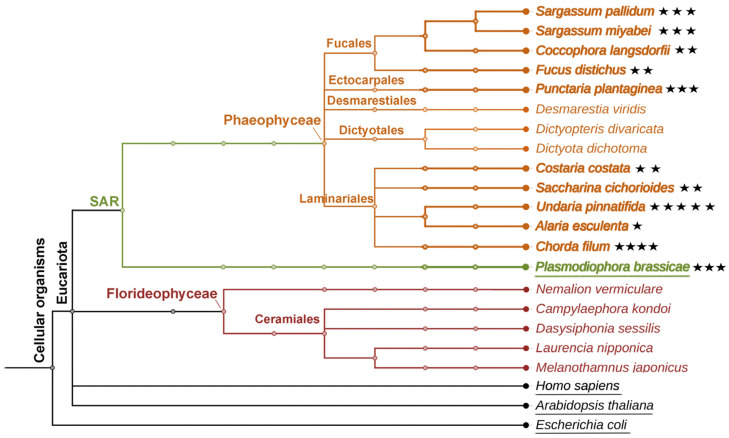
The phylogenetic tree of brown and red algae described in the present study. Occurrence of heterobicyclic oxylipins in algae species is highlighted with asterisks. The number of asterisks corresponds to the number of HPB products in a certain species.

It should be emphasized that HPB products were detected in all of the investigated brown algae species of the orders Ectocarpales, Fucales, and Laminariales (Figure 6). All species of these three orders possessed the epoxycyclopentane, plasmodiophorol A (**4**). In contrast, no HPB products were found in the brown algae of the Desmarestiales and Dictyotales orders or in any of the tested red algae species (Figure 6). Therefore, plasmodiophorol A (**4**) and other HPB products could thus be used as chemotaxonomic markers for the classification of the separate orders of algae within Heterokontophyta.

In contrast to their macrolactone analogs (ecklonialactones) [18,19,20], heterocyclic oxylipins (plasmodiophorols and ectocarpins) have not been detected in Heterokontophyta before the recent work [26]. The first congener of plasmodiophorols and ectocarpins, hybridalactone, was discovered in the red alga *Laurencia hybrida* [17]. Subsequently, the red alga *Agardhiella subulata* was discovered to contain a related compound, agardhilactone [23]. Several analogous heterobicyclic oxylipin derivatives have also been identified in brown algae, including ecklonialactones from *Ecklonia stolonifera* [18,19,20], their haloid derivatives from *Eisenia bicyclis* [21], egregiachlorides from *Egregia menziesii* [22], cymatherol and cymatherolactone [24], as well as cymathere ethers [25] from *Cymathaere triplicata*. The biosynthetic origin of these oxylipins was not experimentally studied until recent works [26,27]. The present study revealed for the first time the widespread occurrence of plasmodiophorols and ectocarpins in Heterokontophyta species belonging to the orders Ectocarpales, Fucales, and Laminariales (Figure 6).

Brown and red algae are rich in compounds exhibiting antioxidant [32,33], anti-inflammatory [34], and immunomodulatory effects [35], for instance, fatty acid derivatives that demonstrate antimicrobial [21] and cytoprotective activities [36]. Additionally, some other biological effects have been revealed for algal oxylipins, for instance, the antifeedant activity of ecklonialactones towards the marine invertebrates [18,19] or the blocking of sodium channels [24]. Understanding the metabolic interactions between different oxylipin pathways in algae could provide insights into stress responses and chemical defense systems in the marine environment.

## 4. Materials and Methods

### 4.1. Materials

Brown algae, *Fucus distichus*, and *Alaria esculenta* thalli were collected on the Barents Sea shore of the Russian Kola Peninsula near Murmansk in July 2025. All other brown and red algal thalli were obtained in July 2013 from the Pacific Institute of Bioorganic Chemistry (Russian Academy of Sciences) marine coastal experimental station near Vladivostok. The brown algae collection included the thalli of *Punctaria plantaginea*, *Coccophora langsdorfii*, *Sargassum miyabei*, *Sargassum pallidum*, *Costaria costata*, *Chorda filum*, *Saccharina cichorioides*, *Undaria pinnatifida*, *Desmarestia viridis*, *Dictyopteris divaricata*, *Dictyota dichotoma*, *Fucus distichus*, and *Alaria esculenta*. The red algal thalli included the species *Dasysiphonia sessilis*, *Campylaephora kondoi*, *Laurencia nipponica*, *Melanothamnus japonicus*, and *Nemalion vermiculare*.

### 4.2. Bioinformatic Methods

The phylogenetic tree was generated using the software PhyloT v2 (https://phylot.biobyte.de/, accessed on 16 September 2025.), a phylogenetic tree generator based on NCBI or the Genome Taxonomy Database, to visualize the evolutionary relationship among species discussed in the present study. The tree was visualized by the iTOL tool (https://itol.embl.de/, accessed on 17 September 2025.).

Model organisms (*Escherichia coli*, *Arabidopsis thaliana*, and *Homo sapiens*), as well as another representative of the SAR group (*P. brassicae*) possessing hydroperoxide bicyclase [27], were used as references for constructing the tree.

### 4.3. Profiling of Oxylipins

The thalli (3 g) were crushed in liquid nitrogen and homogenized with ten volumes (*w*/*v*) of ice-cold hexane-ethyl acetate (1:1) solution. The resulting homogenates were centrifuged at 8000× *g*, 4 °C, for 20 min. The supernatants were decanted and acidified to pH 6.0, and the total lipid extracts were extracted with a 10 mL ethyl acetate-hexane 1:1 (*v*/*v*) solution. The solvent from the resulting mixtures was evaporated. The resultant lipid extracts were dissolved in chloroform-isopropanol 2:1 (*v*/*v*) and filtered via Supelclean LC-NH2 (3 mL) cartridges (Supelco, Merck Group, Darmstadt, Germany). The free carboxylic acids were then eluted with a solvent mixture of ethyl acetate and acetic acid (98:2 *v*/*v*). The products were methylated with ethereal diazomethane and trimethylsilylated with a 1:1:1 (*v*/*v*/*v*) mixture of pyridine, hexamethyldisilazane, and trimethylchlorosilane at 23 °C for 30 min. When specified, the products were reduced with NaBH_4_, methylated, and trimethylsilylated. The silylation reagents were evaporated in a vacuum. Dry residues were dissolved in 100 μL of hexane. The resultant Me esters/TMS derivatives (Me/TMS) were analyzed by GC-MS.

### 4.4. Spectral Studies

The GC-MS studies were performed using a Shimadzu QP5050A mass spectrometer linked to a Shimadzu GC-17A gas chromatograph equipped with a Supelco MDN-5S fused capillary column (length 30 m; ID 0.25 mm; film thickness 0.25 μm). Helium was employed as the carrier gas, with a linear velocity of 30 cm/s. The injections were carried out in split mode, with an initial column temperature of 120 °C and an injector temperature of 230 °C. The temperature was raised linearly at a rate of 10 °C per minute until it reached 240 °C. Electron impact ionization (70 eV) was utilized.

## Figures and Tables

**Table 1:** Endogenous abundance of oxylipins (Me/TMS) in some algae. Total oxylipins (free acids) upon extraction from algal thalli were purified with the RP and aminopropyl solid-phase extraction (SPE) cartridges, methylated, trimethylsylilated and analyzed by GC-MS in Materials and Methods.

Clade	Order	Species	Detected Compound Numbers												
			1	2	3	4	5	6	7	8	9	10	11	δ-Ketols	Triols
Heterokontophyta	Ectocarpales	***Punctaria plantaginea***				**+**	**+**	**+**	**+**		**+**	**+**			
	Fucales	***Coccophora langsdorfii***				**+**		**+**				**+**		**+**	**+**
		***Fucus distichus***	**+**	**+**	**+**	**+**		**+**	**+**					**+**	**+**
		***Sargassum miyabei***	**+**		**+**	**+**		**+**				**+**	**+**		
		***Sargassum pallidum***	**+**		**+**	**+**		**+**			**+**			**+**	**+**
	Laminariales	***Alaria esculenta***	**+**	**+**		**+**									
		***Costaria costata***				**+**		**+**	**+**			**+**			
		***Chorda filum***		**+**		**+**		**+**	**+**	**+**	**+**	**+**		**+**	**+**
		***Saccharina cichorioides***	**+**		**+**	**+**	**+**				**+**	**+**	**+**	**+**	**+**
		***Undaria pinnatifida***		**+**	**+**	**+**	**+**	**+**	**+**	**+**	**+**	**+**	**+**	**+**	**+**
	Desmarestiales	*Desmarestia viridis*			**+**									**+**	**+**
	Dictyotales	*Dictyopteris divaricata*													**+**
		*Dictyota dichotoma*													**+**
Rhodophyta	Ceramiales	*Dasysiphonia sessilis*													**+**
		*Campylaephora kondoi*			**+**									**+**	**+**
		*Laurencia nipponica*	**+**		**+**									**+**	**+**
		*Melanothamnus japonicus*			**+**									**+**	**+**
	Nemaliales	*Nemalion vermiculare*	**+**		**+**									**+**	**+**

## Data Availability

No data was used for the research described in the article.

## Supplementary Materials

The following supporting information can be downloaded at: https://www.mdpi.com/article/10.3390/md24010008/s1. Figure S1: GC-MS profiles (TIC, total ion current chromatograms) of the endogenous oxylipins (Me/TMS) from thalli of *Undaria pannatiifida* (**A**) and *Chorda filum* (**B**). 22:0 and 24:0, peaks of docosanoic and tetracosanoic acids (Me), respectively. 13-HOT, (9*Z*,11*E*,15*Z*)-13-hydroxy-9,11,15-octadecatrienoic acid (Me/TMS). 9-HOD, (10*E*,12*Z*)-9-hydroxy-10,12-octadecadienoic acid (Me/TMS). 13-HOD, (9*Z*,11*E*)-13-hydroxy-9,11-octadecadienoic acid (Me/TMS). Compound **8**, ectocarpin A, 3-[(1′*E*)-propenyl]-6-oxabicyclo[3.1.0]hexane. Compound **7**, α-ketol 12-oxo-13-hydroxy-9-octadecenoic acid (Me/TMS). Compound **9**, plasmodiophorol B. Compound **4**, plasmodiophorol A. Compound **6**, plasmodiophorol C. Compound **10**, ectocarpin C. Compound **11**, ectocarpin D. EAlc, epoxyalcohols. EAlc, stereoisomeric epoxyalcohols, 11-hydroxy-12,13-epoxy-9-octadecenoic acid (Me/TMS); Figure S2: Mass spectra and fragmentation schemes (insets) for compounds **4**, **6**, **8**, and **9** (plasmodiophorol A, plasmodiophorol C, ectocarpin A, and plasmodiophorol B, respectively); Figure S3: Mass spectra and fragmentation schemes (insets) for compounds **10** and **11** (ectocarpin C and ectocarpin D, respectively).

## Abbreviations

The following abbreviations are used in this manuscript:

HPB	hydroperoxide bicyclase
EAS	epoxyalcohol synthase
AOS	allene oxide synthase
9-HOD	(9*S*,10*E*,12*Z*)-9-hydroxy-10,12-octadecadienoic acid
13-H(P)OD	(9*Z*,11*E*,13*S*)-13-hydro(pero)xy-9,11-octadecadienoic acid
13-H(P)OT	(9*Z*,11*E*,13*S*,15*Z*)-13-hydro(pero)xy-9,11,15-octadecatrienoic acid
GC-MS	gas chromatography—mass-spectrometry
Me	methyl
TMS	trimethylsilyl
TIC	total ion current
SIC	selected ion current

## References

[1] Calder P.C. (2020). Eicosanoids. Essays Biochem..

[2] Grechkin A.N. (1998). Recent developments in biochemistry of the plant lipoxygenase pathway. Prog. Lipid Res..

[3] Andreou A., Brodhun F., Feussner I. (2009). Biosynthesis of oxylipins in non-mammals. Prog. Lipid Res..

[4] Brash A.R. (2009). Mechanistic aspects of CYP74 allene oxide synthases and related cytochrome P450 enzymes. Phytochemistry.

[5] Lee D.S., Nioche P., Hamberg M., Raman C.S. (2008). Structural insights into the evolutionary paths of oxylipin biosynthetic enzymes. Nature.

[6] Grechkin A.N., Hamberg M. (2004). The “heterolytic hydroperoxide lyase” is an isomerase producing a short-lived fatty acid hemiacetal. Biochim. Biophys. Acta.

[7] Grechkin A.N., Brühlmann F., Mukhtarova L.S., Gogolev Y.V., Hamberg M. (2006). Hydroperoxide lyases (CYP74C and CYP74B) catalyze the homolytic isomerization of fatty acid hydroperoxides into hemiacetals. Biochim. Biophys. Acta.

[8] Mukhtarova L.S., Brühlmann F., Hamberg M., Khairutdinov B.I., Grechkin A.N. (2018). Plant hydroperoxide-cleaving enzymes (CYP74 family) function as hemiacetal synthases: Structural proof of hemiacetals by NMR spectroscopy. Biochim. Biophys. Acta.

[9] Grechkin A.N. (2002). Hydroperoxide lyase and divinyl ether synthase. Prostaglandins Other Lipid Mediat..

[10] Hamberg M. (2005). Hidden stereospecificity in the biosynthesis of divinyl ether fatty acids. FEBS J..

[11] Toporkova Y.Y., Fatykhova V.S., Gogolev Y.V., Khairutdinov B.I., Mukhtarova L.S., Grechkin A.N. (2017). Epoxyalcohol synthase of *Ectocarpus siliculosus*. First CYP74-related enzyme of oxylipin biosynthesis in brown algae. Biochim. Biophys. Acta.

[12] Toporkova Y.Y., Smirnova E.O., Gorina S.S., Mukhtarova L.S., Grechkin A.N. (2018). Detection of the first higher plant epoxyalcohol synthase: Molecular cloning and characterisation of the CYP74M2 enzyme of spikemoss *Selaginella moellendorffii*. Phytochemistry.

[13] Proteau P.J., Gerwick W.H. (1993). Divinyl ethers and hydroxy fatty acids fromthree species of Laminaria (brown algae). Lipids.

[14] Jiang Z.D., Gerwick W.H. (1997). Novel oxylipins from the temperate red alga *Polyneura latissima*: Evidence for an arachidonate 9(*S*)-lipoxygenase. Lipids.

[15] Gerwick W.H. (1993). Carbocyclic oxylipins of marine origin. Chem. Rev..

[16] Barbosa P., Valentão M., Andrade P.B. (2016). Biologically active oxylipins from enzymatic and nonenzymatic routes in macroalgae. Mar. Drugs.

[17] Higgs M.D., Mulheirn L.J. (1981). Hybridalactone, an unusual fatty acid metabolite from the red alga *Laurencia hybrida* (Rhodophyta, Rhodomelaceae). Tetrahedron.

[18] Kurata K., Taniguchi K., Shiraishi K., Hayama N., Tanaka I., Suzuki M. (1989). Ecklonialactone-A and-B, two unusual metabolites from the brown alga *Eckloniastolonifera* Okamura. Chem. Lett..

[19] Kurata K., Taniguchi K., Shiraishi K., Suzuki M. (1993). Ecklonialactones-C-F from the brown alga *Ecklonia stolonifera*. Phytochemistry.

[20] Todd J.S., Proteau P.J., Gerwick W.H. (1994). The absolute configuration of ecklonialactones A, B, and E, novel oxylipins from brown algae of the genera *Ecklonia* and *Egregia*. J. Nat. Prod..

[21] Kousaka K., Ogi N., Akazawa Y., Fujieda M., Yamamoto Y., Takada Y., Kimura J. (2003). Novel oxylipin metabolites from the brown alga *Eisenia bicyclis*. J. Nat. Prod..

[22] Todd J.S., Proteau P.J., Gerwick W.H. (1993). Egregiachlorides A-C: New chlorinated oxylipins from the marine brown alga *Egregia menziesii*. Tetrahedron Lett..

[23] Graber M.A., Gerwick W.H., Cheney D.P. (1996). The isolation and characterization of agardhilactone, a novel oxylipin from the marine red alga *Agardhiella subulata*. Tetrahedron Lett..

[24] Choi H., Proteau P.J., Byrum T., Gerwick W.H. (2012). Cymatherelactone and cymatherols A-C, polycyclic oxylipins from the marine brown alga *Cymathere triplicate*. Phytochemistry.

[25] Proteau P.J., Gerwick W.H. (1992). Cymathere ethers A and B: Bicyclic oxylipins from the marine brown alga *Cymathere triplicate*. Tetrahedron Lett..

[26] Toporkova Y.Y., Smirnova E.O., Mukhtarova L.S., Grechkin A.N. (2022). Lipoxygenase pathway in brown algae: The biosynthesis of novel oxylipins ‘ectocarpins’ by hydroperoxide bicyclase CYP5164A3 of *Ectocarpus siliculosus*. Biochim. Biophys. Acta.

[27] Grechkin A.N., Lantsova N.V., Khairutdinov B.I., Toporkova Y.Y. (2021). Hydroperoxide bicyclase CYP50918A1 of *Plasmodiophora brassicae* (Rhizaria, SAR): Detection of novel enzyme of oxylipin biosynthesis. Biochim. Biophys. Acta.

[28] Grechkin A.N., Kuramshin R.A., Latypov S.K., Safonova Y.Y., Gafarova T.E., Ilyasov A.V. (1991). Hydroperoxides of alpha-ketols. Novel products of the plant lipoxygenase pathway. Eur. J. Biochem..

[29] Hamberg M. (1991). Transformations of alpha-linolenic acid in leaves of corn (*Zea mays* L.). Adv. Prostaglandin Thromboxane Leukotriene Res..

[30] Hamberg M. (1987). Mechanism of corn hydroperoxide isomerase: Detection of 12,13(*S*)-oxido-9-(*Z*),11-octadecadienoic acid. Biochim. Biophys. Acta.

[31] Thomas C.P., Boeglin W.E., Garcia-Diaz Y., O’Donnell V.B., Brash A.R. (2013). Steric analysis of epoxyalcohol and trihydroxy derivatives of 9-hydroperoxy-linoleic acid from hematin and enzymatic synthesis. Chem. Phys. Lipids.

[32] Catarino M.D., Silva-Reis R., Chouh A., Silva S., Braga S.S., Silva A.M.S., Cardoso S.M. (2023). Applications of antioxidant secondary metabolites of *Sargassum* spp.. Mar. Drugs.

[33] Jagtap A.S., Singh A., Jain A. (2025). Extraction, structural characterization, and bioactivity of laminarin and fucoidan from brown macroalgae *Chorda filum*. Carbohydr. Res..

[34] Kim O.-K., Lee M., Kwon H.O., Lee D., Park J., Kim E., You Y., Lim Y.T., Jun W., Lee J. (2018). *Costaria costata* extract suppresses development of atopic dermatitis in chloro-2,4-dinitrobenzene-treated NC/Nga Mice. Skin. Pharmacol. Physiol..

[35] Cheong K.-L., Chen W., Wang M., Zhong S., Veeraperumal S. (2025). Therapeutic prospects of *Undaria pinnatifida* polysaccharides: Extraction, purification, and functional Activity. Marine Drugs.

[36] Wells M.L., Potin P., Craigie J.S., Raven J.A., Merchant S.S., Helliwell K.E., Smith A.G., Camire M.E., Brawley S.H. (2017). Algae as nutritional and functional food sources: Revisiting our understanding. J. Appl. Phycol..

