# Supplementary material for: Oxylipin Profiling in Selected Brown and Red Algae: Detection of Heterobicyclic Oxylipins, Plasmodiophorols and Ectocarpins in Phaeophyceae
Source: Mar Drugs. 2025 Dec 23;24(1):8. doi: 10.3390/md24010008 (PMC12843491; doi:10.3390/md24010008)
Supplement: Supplementary file 1 [file marinedrugs-24-00008-s001.zip › marinedrugs-4017068-supplementary.pdf]

Supplementary Information

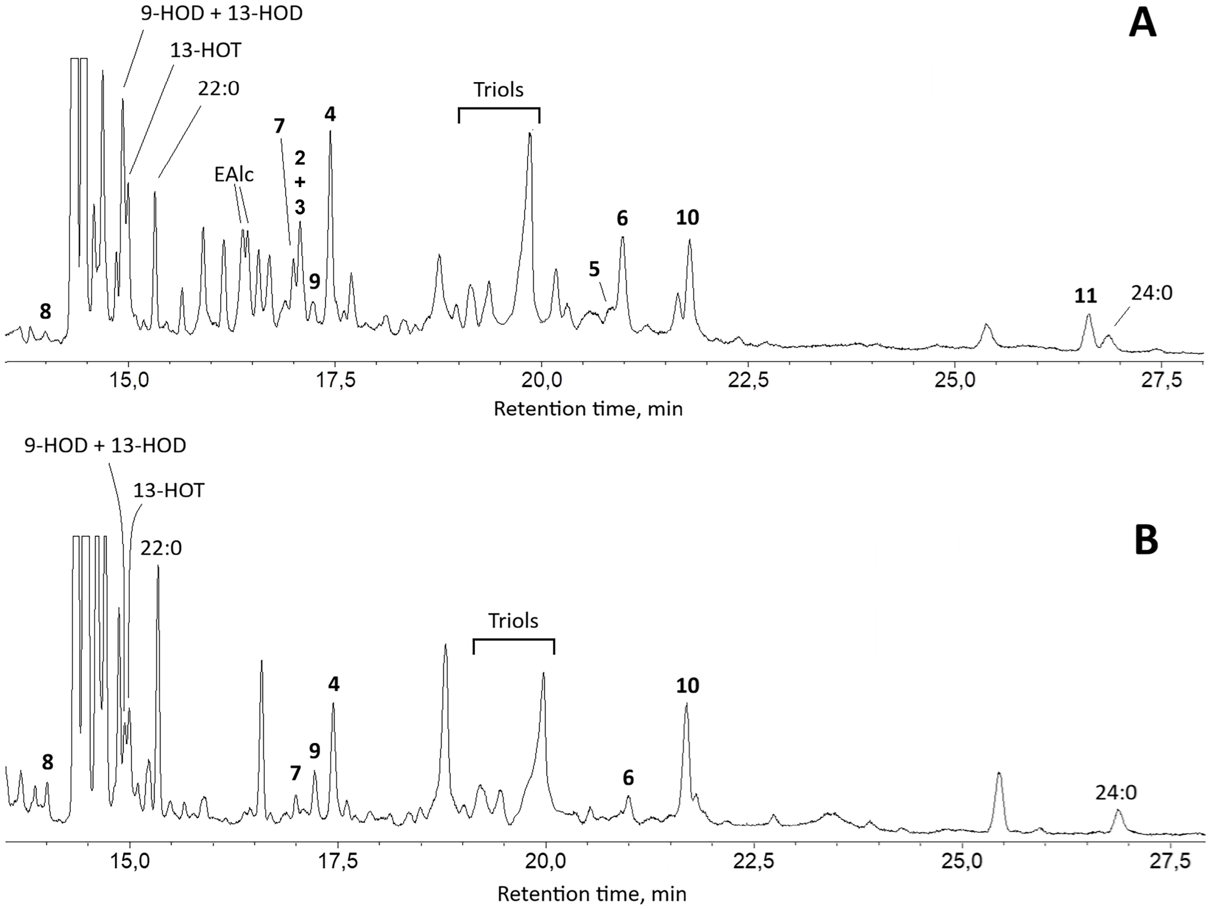

Supplementary Figure S1. GC-MS profiles (TIC, total ion current chromatograms) of the endogenous oxylipins (Me/TMS) from thalli of *Undaria pannatiifida* (**A**) and *Chorda filum* (**B**). 22:0 and 24:0, peaks of docosanoic and tetracosanoic acids (Me), respectively. 13-HOT, (9*Z*,11*E*,15*Z*)-13-hydroxy-9,11,15-octadecatrienoic acid (Me/TMS). 9-HOD, (10*E*,12*Z*)-9-hydroxy-10,12-octadecadienoic acid (Me/TMS). 13-HOD, (9*Z*,11*E*)-13-hydroxy-9,11-octadecadienoic acid (Me/TMS). Compound **8**, ectocarpin A, 3-[(1′*E*)-propenyl]-6-oxabicyclo[3.1.0]hexane. Compound **7**, α-ketol 12-oxo-13-hydroxy-9-octadecenoic acid (Me/TMS). Compound **9**, plasmodiophorol B. Compound **4**, plasmodiophorol A. Compound **6**, plasmodiophorol C. Compound **10**, ectocarpin C. Compound **11**, ectocarpin D. EAlc, epoxyalcohols. EAlc, stereoisomeric epoxyalcohols, 11-hydroxy-12,13-epoxy-9-octadecenoic acid (Me/TMS).

4

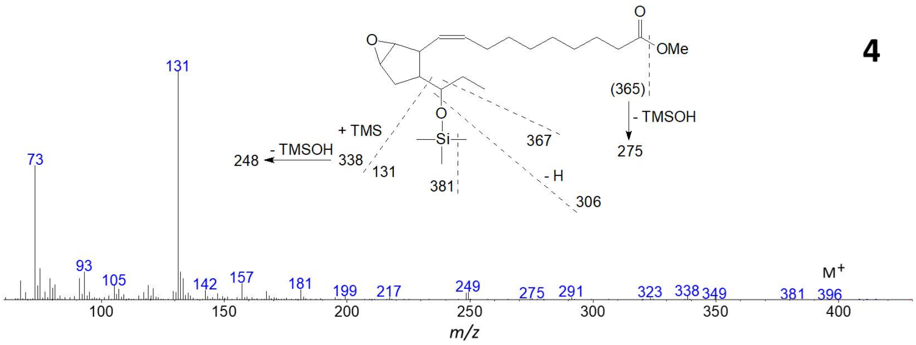

6

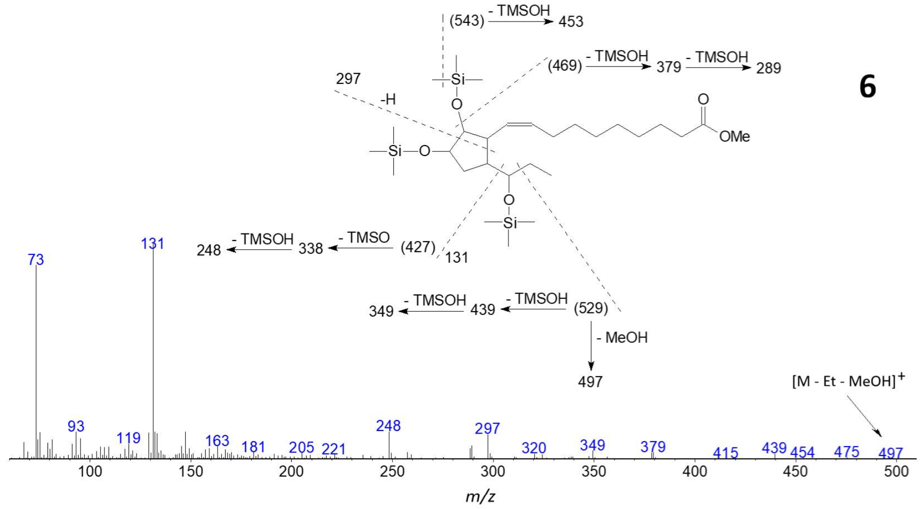

8

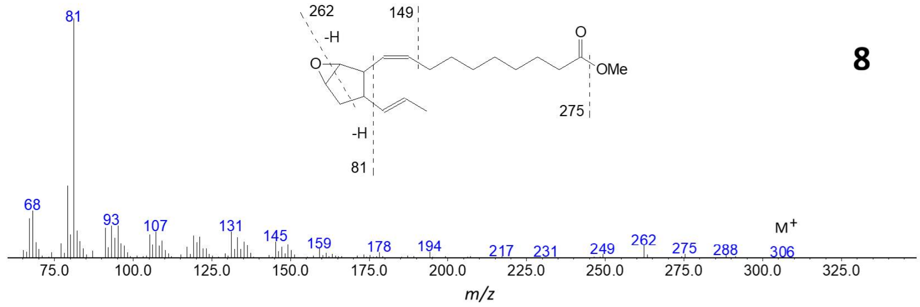

9

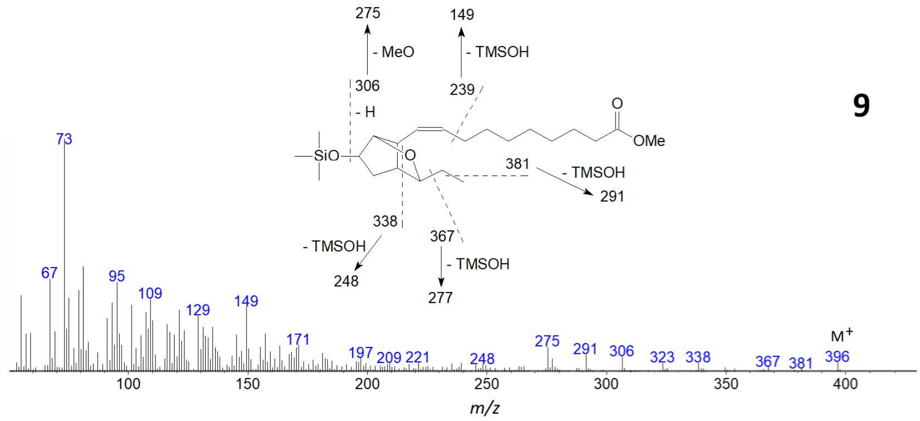

Supplementary Figure S2. Mass spectra and fragmentation schemes (insets) for compounds **4, 6, 8,** and **9** (plasmodiophorol A, plasmodiophorol C, ectocarpin A, and plasmodiophorol B, respectively).

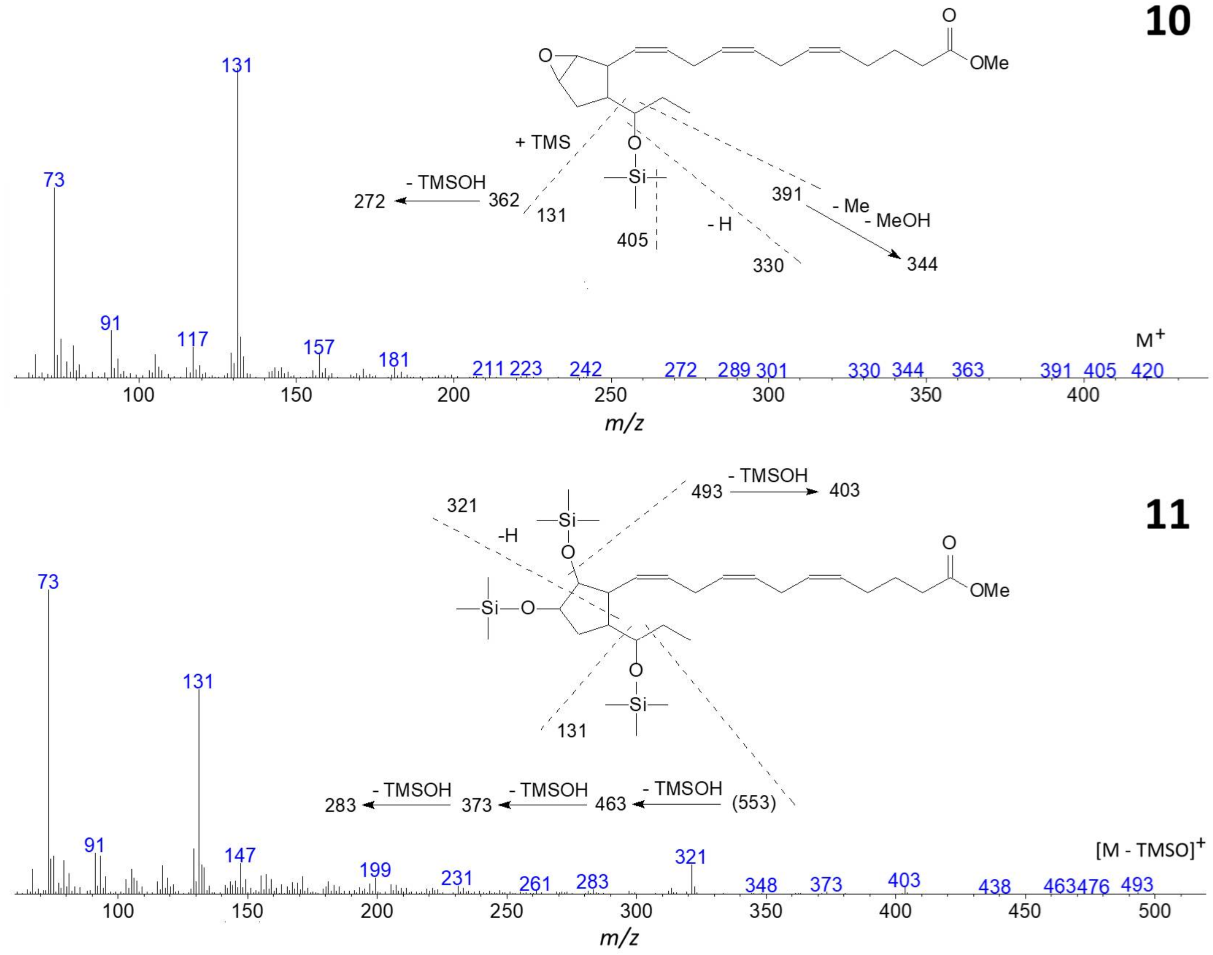

Supplementary Figure S3. Mass spectra and fragmentation schemes (insets) for compounds **10** and **11** (ectocarpin C and ectocarpin D, respectively).
